# Oxidized Low-Density Lipoprotein Induces Reactive Oxygen Species-Dependent Proliferation of Intestinal Epithelial Cells

**DOI:** 10.3390/ph17111466

**Published:** 2024-11-01

**Authors:** Eddy E. Gonzalez-Horta, Juan F. Burgueno, María J. Leiva, Carla Villavicencio, Fernando I. Kawaguchi, Hajar Hazime, Fátima Reyes, Viana Manrique-Suárez, Natalie C. Parra, Maria T. Abreu, Jorge R. Toledo

**Affiliations:** 1 Biotechnology and Biopharmaceutical Laboratory, Departamento de Fisiopatología, Facultad de Ciencias Biológicas, Universidad de Concepción, Víctor Lamas 1290, P.O. Box 160-C, Concepción 4030000, Chile; eddygonzalez@udec.cl (E.E.G.-H.); mariajleiva@udec.cl (M.J.L.); cvillavicen2019@udec.cl (C.V.); fatima8526@gmail.com (F.R.); vianamanriquesuarez@gmail.com (V.M.-S.); natparra@udec.cl (N.C.P.); 2 Division of Gastroenterology, Department of Medicine, University of Miami–Miller School of Medicine, Miami, FL 33136, USA; joan.burgueno@gmail.com (J.F.B.); h.hazime1@umiami.edu (H.H.); mabreu1@med.miami.edu (M.T.A.); 3 EusmedicaL Medical Centre, Concepción 4070274, Chile; itarokawaguchi@vtr.net

**Keywords:** oxidized low-density lipoprotein, human colonoids, reactive oxygen species, proliferation

## Abstract

**Background/Objectives**: Oxidized low-density lipoprotein (ox-LDL) is a proinflammatory particle associated with various diseases and affects cell proliferation and viability in multiple cell types. However, its impact on intestinal epithelial cells remains underexplored. This study investigates the effect of ox-LDL on colonic epithelial cell proliferation and viability, as well as the underlying mechanisms involved. **Methods**: The expression levels of ox-LDL receptors in human colonoids were analyzed at baseline and in response to proinflammatory signals by qRT-PCR. The effect of ox-LDL on organoid proliferation was analyzed using morphometric measurements, viability assays, and the incorporation of a thymidine analog into DNA. The generation of reactive oxygen species (ROS) was determined by Amplex Red assays. Additionally, ox-LDL-induced ROS-dependent organoid proliferation was studied by exposing colonoids to an antioxidant or ROS inhibitors. **Results**: Colonic epithelial cells express ox-LDL receptors. Ox-LDL significantly induces the proliferation of colonic epithelial cells, which are dependent on ROS generation. Notably, ROS scavengers and NADPH inhibitors reduced ox-LDL-induced proliferation, highlighting the crucial role of oxidative stress in this process. **Conclusions**: This study demonstrates for the first time that ox-LDL stimulates CEC proliferation mediated by ROS production and validates that the colonic organoid model enables the analysis of potential pharmacological strategies for intestinal diseases characterized by oxidative stress and inflammation.

## 1. Introduction

In chronic inflammatory diseases, the underlying oxidative stress can cause lipoprotein oxidation, such as low-density lipoprotein (LDL), producing oxidized LDL (ox-LDL). Ox-LDL is more atherogenic and proinflammatory than LDL, causing endothelial dysfunction and cardiovascular conditions. However, its role extends beyond the cardiovascular system, affecting cell proliferation and viability across different cell types [1]. Ox-LDL is mainly removed from circulation by scavenger receptors such as the CD36 molecule (CD36), macrophage scavenger receptor 1 (MSR1), and oxidized low-density lipoprotein receptor 1 (LOX-1) [2]. The effects of ox-LDL on cell biology are complex and vary depending on the concentration and cell type involved [1,3].

Cellular uptake of ox-LDL induces oxidative stress in various cell types, increasing reactive oxygen species (ROS) production [4,5]. Ox-LDL uptake by macrophages and endothelial cells significantly elevates intracellular ROS levels [6,7,8]. This increase in ROS is linked to NADPH oxidase (NOX) activation and the subsequent signaling pathways that mediate inflammatory responses [7,9]. While much is known about these mechanisms in vascular tissues, the way ox-LDL affects intestinal epithelial cells remains underexplored.

The intestinal epithelium is a key barrier in maintaining gut homeostasis, but it can be disrupted in inflammatory bowel disease (IBD). Inflammation in these conditions creates a microenvironment rich in oxidative stress, potentially facilitating the oxidation of LDL and increasing the local concentration of ox-LDL.

Organoids are three-dimensional in vitro cultures that can closely mimic the structural and functional characteristics of their tissue of origin, making them invaluable tools for studying disease mechanisms and drug responses. Organoids derived from colorectal tissues have gained prominence as a preclinical model for investigating colorectal cancer biology and testing pharmacological treatments [10]. Their ability to recapitulate the genetic and phenotypic heterogeneity of colorectal tumors makes them especially suitable for evaluating the efficacy and safety of anticancer agents, allowing researchers to explore therapeutic responses in a controlled and physiologically relevant environment [10,11].

Given their advantages, organoids represent a promising platform for developing personalized treatment strategies for colorectal cancer, enabling the screening of compounds that could potentially target cancer stem cells or disrupt specific signaling pathways involved in tumor progression [11,12]. By utilizing this advanced model, we aimed to explore how ox-LDL influences the proliferation of colonic epithelial cells and assess the potential implications for colorectal cancer therapeutics. In this study, we investigated the expression of ox-LDL receptors in human colonoids and evaluated organoid growth, cellular proliferation, and ROS formation upon exposure to ox-LDL.

## 2. Results

**Figure 1:**
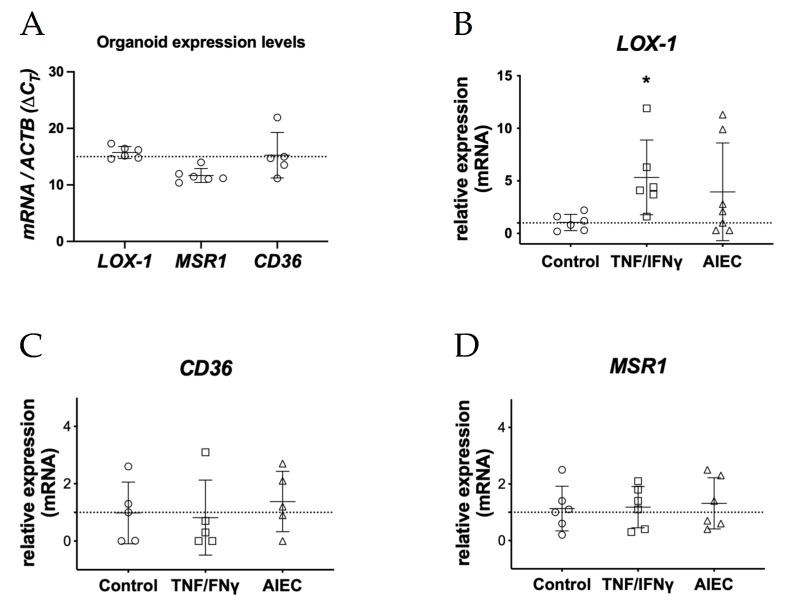
Expression of scavenger receptors in colonic epithelial cells. (**A**) mRNA expression of ox-LDL receptors in colonoids in basal conditions. Note that lower ΔCt values mean higher expression levels. (**B**) Proinflammatory signals induce *OLR1* (LOX-1) expression but not *CD36* (**C**) or *MSR1* (**D**). Kruskall–Wallis test and Dunn’s multiple comparisons test, * *p* < 0.05.

We aimed to characterize the expression of the scavenger receptors that mediate ox-LDL recognition and internalization in human colonic organoids. Colonoids were collected under steady conditions and stimulated with proinflammatory stimuli to assess the mRNA expression of oxidized low-density lipoprotein receptor 1 (*ORL1*), *CD36*, and *MSR1* transcripts through quantitative polymerase chain reaction. At baseline, we identified *MSR1* as a gene with intermediate expression levels in human colonoids, while *CD36* and *ORL1* exhibited intermediate-low expression levels (Figure 1A).

Inflammatory signals could increase the expression of scavenger receptors [13,14,15]. We compared the expression of these ox-LDL receptors in response to proinflammatory stimuli, specifically tumor necrosis factor plus interferon-gamma (TNFα/IFNγ) and heat-killed adherent-invasive *E. coli* (AIEC). Colonoids significantly upregulated *OLR1* expression compared with untreated controls. However, no expression changes were detected for *CD36* or *MSR1* under these conditions (Figure 1B). These findings show that colonic epithelial cells (CEC) express specific ox-LDL receptors and overexpress LOX-1 in response to proinflammatory and bacterial cues, and they may internalize ox-LDL in colonic inflammatory conditions.

**Figure 2:**
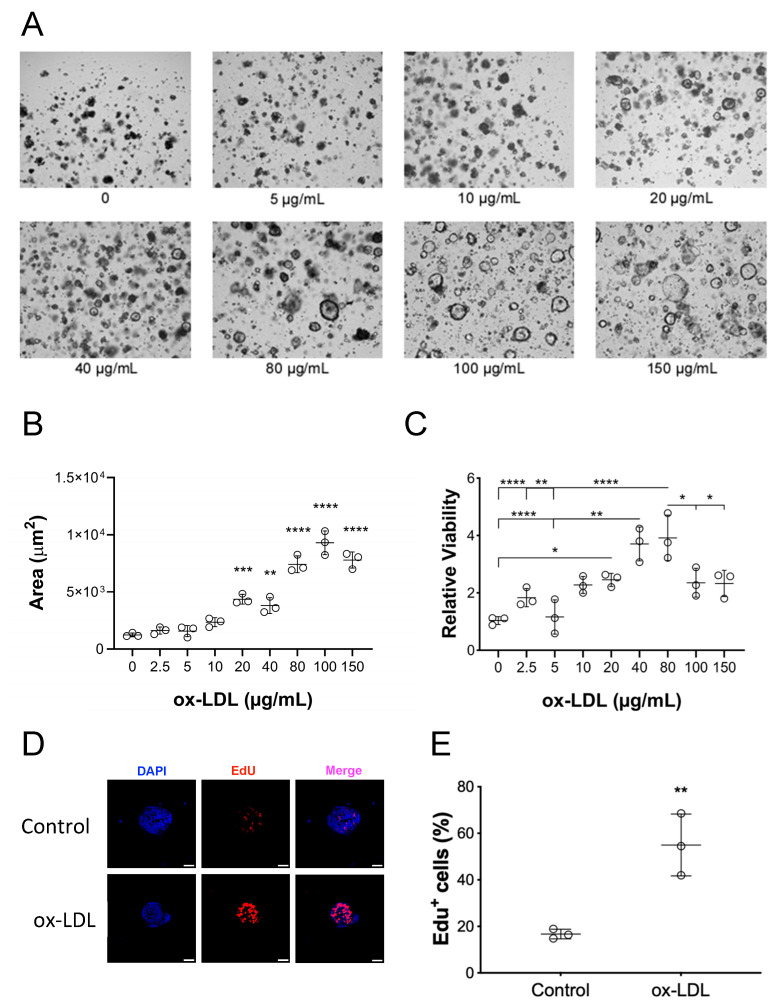
Ox-LDL induces the growth of human colonoids. (**A**) Organoids were treated with increasing concentrations of ox-LDL for 4 days. Contrast photomicrographs, 4× magnification, scale bar: 100 μm. (**B**) Quantification of organoid surface area (n = 3). One-way ANOVA and Tukey’s multiple comparisons test, ** *p* < 0.01; *** *p* < 0.001; **** *p* < 0.0001 vs. 0; 2.5; 5; and 10 μg/mL of ox-LDL. (**C**) Viability of organoids. Each condition was analyzed in duplicate (n = 3). One-way ANOVA and Tukey’s multiple comparisons test, * *p* < 0.05; ** *p* < 0.01; **** *p* < 0.0001. (**D**) EdU incorporation in colonoids treated with ox-LDL. Representative images of organoids incubated with 80 μg/mL of ox-LDL. DNA was stained with DAPI. Images are representative of three independent experiments. Scale bar: 50 μm. (**E**) Quantification of EdU+ cells. Mann–Whitney test, ** *p* < 0.01.

To investigate the impact of ox-LDL in CEC growth, colonoids were exposed to varying concentrations of ox-LDL, ranging from 20 to 150 µg/mL, and their size and viability were assessed. The average size of colonoids, as well as their viability, significantly increased in a dose–response manner and plateaued at 80–100 µg/mL of ox-LDL (Figure 2A–C). To confirm that the increase in size and viability of ox-LDL-treated organoids was due to cell proliferation, we analyzed the incorporation of the thymidine analog 5-ethyl-2′-deoxyuridine (EdU) into DNA. After a 2 h pulse with EdU, the colonoids challenged with ox-LDL showed more proliferating cells than those treated with vehicle (Figure 2D). These findings indicate that ox-LDL stimulates CEC proliferation.

**Figure 3:**
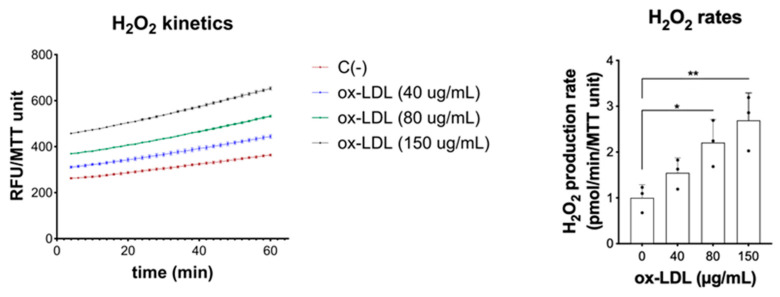
Ox-LDL induces epithelial production of reactive oxygen species and colonoid production of H_2_O_2_. The left panel shows the kinetic production of H_2_O_2_ in one representative experiment. The right panel shows the H_2_O_2_ production rate in all experiments (n = 3). Data were analyzed by One-way ANOVA, followed by Dunnett´s post hoc test, * *p* < 0.05; ** *p* < 0.01.

Ox-LDL can induce the generation of reactive oxygen species (ROS) in different cell types [16] via NADPH oxidase activation, mitochondrial production, and secondary lipid peroxidation [17]. To investigate whether ROS can be primarily produced in CEC in response to ox-LDL, we examined H_2_O_2_ production in colonoids stimulated with this modified lipoprotein. Human colonoids exposed to increasing concentrations of ox-LDL showed a significant dose-dependent increase in the production rate of H_2_O_2_ as compared with unstimulated colonoids (Figure 3).

**Figure 4:**
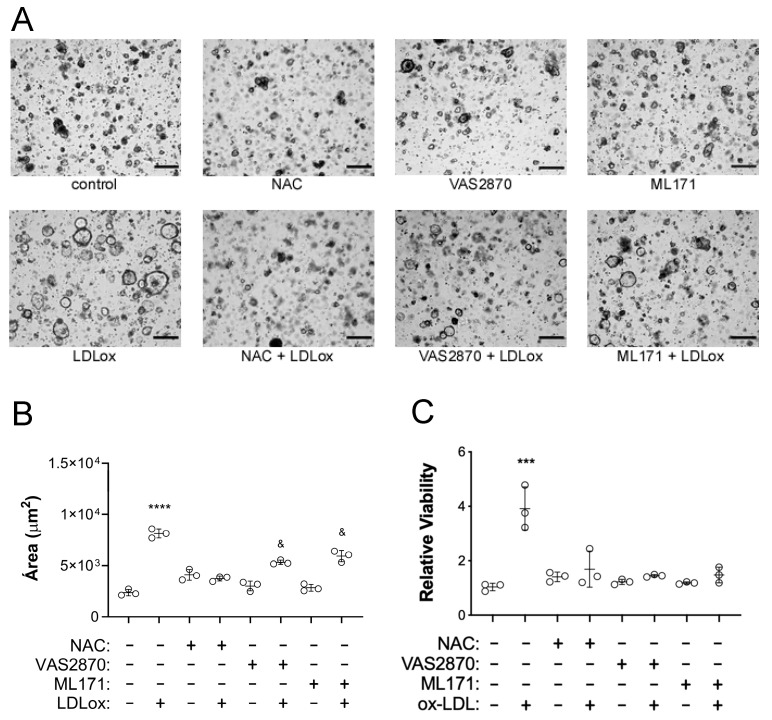
Human ox-LDL-dependent colonoid growth is mediated by ROS. (**A**) Colonoids were incubated with 80 µg/mL of ox-LDL with/without 10 µM VAS2870, 0.25 µM ML171, or 2.5 mM NAC for 4 days. Contrast photomicrographs, 4× magnification, scale bar: 100 μm. (**B**) Quantification of organoid surface area from 3 independent experiments. One-way ANOVA and Tukey’s multiple comparisons test, **** *p* < 0.0001 vs. all groups, and *p* < 0.001 vs. control, VAS2870, and ML171. (**C**) Viability of organoids. Each condition was analyzed in duplicate (n = 3). One-way ANOVA and Tukey’s multiple comparisons test, *** *p* < 0.001 vs. all groups.

ROS can favor the proliferation of CEC [18]. To determine whether ox-LDL-dependent growth and viability of human colonoids were influenced by ox-LDL-mediated ROS production, colonoids were stimulated with 80 μg/mL of ox-LDL along with 10 µM of VAS2870 (a pan-inhibitor of NADPH oxidases), 0.25 µM of ML171 (an inhibitor of the NADPH oxidase NOX1), or 2.5 mM of N-acetylcysteine (NAC), a non-specific ROS scavenger [19]. We found that ROS inhibition significantly arrested colonoid growth compared with colonoids stimulated with ox-LDL alone (Figure 4A–C). NAC caused the most remarkable arrest in CEC growth, completely abrogating the effects of ox-LDL on colonoid size and viability. Conversely, the size of colonoids treated with ox-LDL along VAS2870 and ML171 was only partially reduced (Figure 4C). These findings suggest that the source of ROS in colonoids stimulated by ox-LDL is not limited to NADPH oxidase activity but may also trigger alternative intracellular sources of ROS.

## 3. Discussion

The oxidation of low-density lipoproteins produces a particle that is no longer recognized by the LDL receptor [20]. Instead, this modified lipoprotein is now recognized by a group of receptors known as scavenger receptors [21]. Oxidized LDL has been implicated in various pathological processes, including atherosclerosis, cancer, and endothelial dysfunction; however, its role extends beyond the cardiovascular system, affecting cell proliferation and viability across different cell types [1,21].

Little is known about the biological regulation of ox-LDL receptors in colon epithelial cells. On the other hand, cytokines like TNFα and pathogen-associated molecular pattern molecules such as LPS upregulate their expression in kidney and lung epithelial cells [22,23,24]. These findings suggest that inflammation may modulate the expression of these receptors in colonic epithelial cells. In this study, we show that human colonic epithelial cells express the ox-LDL receptors CD36, MSR1, and LOX-1. These findings are consistent with previous studies reporting the expression of scavenger receptors in small and large intestinal epithelial cells [25,26,27]. Additionally, we found that LOX-1 expression increases upon exposure to proinflammatory stimuli. In the context of IBD, the presence of proinflammatory cytokines and bacterial antigens can lead to the altered expression of scavenger receptors, which may enhance the uptake of ox-LDL and exacerbate inflammatory responses.

Notably, our findings indicate that colonoids can tolerate relatively high concentrations of ox-LDL without adversely affecting their growth and viability. Previous studies have shown that low or moderate concentrations of ox-LDL can promote cell proliferation (less than 50 µg/mL). In contrast, higher concentrations can be toxic or lead to cell cycle arrest. In prostate cancer lines, ox-LDL promoted proliferation at concentrations between 10 and 100 µg/mL [28]. However, ox-LDL significantly inhibited the proliferation of leukemia esophageal carcinoma cells at 20 µg/mL [1]. Additionally, ox-LDL stimulated proliferation in umbilical cord endothelial cells at concentrations lower than 40 µg/mL but inhibited proliferation at higher concentrations [1]. Human fibroblast growth was also stimulated by concentrations of ox-LDL between 10 and 50 µg/mL [29,30]. However, ox-LDL did not stimulate proliferation and instead had a cytotoxic effect in some cancer cells, including the colorectal cancer cells HT29, the ovarian cancer cells OVCAR3 and OVCAR5, and the lung cancer cells A549 [31]. Colonoid tolerability to high concentrations of ox-LDL can be explained because these three-dimensional structures are embedded in an extracellular matrix that can restrict the diffusion of the ox-LDL particles, modulating the toxicity of ox-LDL [32].

To our knowledge, this is the first report demonstrating an ox-LDL-driven proliferative effect in CEC. This effect is potentially mediated by ox-LDL receptors, which were expressed in CEC. Specifically, LOX-1 was upregulated in response to a proinflammatory challenge in our in vitro setup and has been associated with tumor progression [33,34]. LOX-1 upregulation by proinflammatory stimuli suggests a mechanism through which ox-LDL may influence epithelial cell behavior. Notably, ox-LDL can also act as a damage-associated molecular pattern, stimulating intracellular signaling pathways through other receptors such as Toll-like receptor 4 [35], a known activator of WNT signaling in CEC [36]. Mechanistically, activation of these receptors may induce the primary production of ROS in CEC [19], ultimately leading to epithelial proliferation and growth. Indeed, redox signaling can activate various intracellular signaling pathways that increase cell proliferation, enhance cell survival, and/or stimulate the production of proinflammatory molecules, favoring a tumor-promoting microenvironment [37], particularly in the context of IBD.

Although IBD is characterized by an increased oxidative stress, there are conflicting results regarding ox-LDL levels. Some studies reported increased levels of ox-LDL and antibodies against ox-LDL, but others reported low levels of ox-LDL, probably because IBD patients frequently have hypocholesterolemia [38,39,40]. However, plasmatic thiobarbituric acid-reactive substances, a marker of lipid peroxidation, were increased in these and other studies [39,41,42]. Serum ox-LDL levels may not reflect the local concentration at inflamed sites. This discrepancy may be attributed to the increased permeability of the intestinal microvascular system in IBD [43,44]. It is considered that lipoprotein extravasation and retention in the extracellular matrix space is increased, and LDL retention by proteoglycans promotes the oxidative modifications of LDL particles [45]. Additionally, the oxidative environment caused by inflammation could favor LDL oxidation. Oxidative modifications of LDL occur upon exposure to ROS, such as superoxide anion, hydrogen peroxide, and myeloperoxidase products [46]. Therefore, the existence of a chronic inflammatory state will serve as fuel to accelerate oxidative modifications of LDL.

Our findings highlight the importance of ox-LDL as a promoter of cellular proliferation and its role in potentially favoring tumor development in the context of chronic inflammatory bowel diseases. Given that oxidative stress markers are elevated in patients with IBD, ox-LDL may play a relevant role in promoting a microenvironment favorable to colon tumorigenesis. The generation of ROS by ox-LDL suggests that the modulation of redox signaling pathways could be a feasible therapeutic target to control epithelial cell proliferation mediated by ox-LDL.

The use of the colonoid model in this study provides a robust and physiologically relevant platform for investigating the effects of ox-LDL and other proinflammatory stimuli on the proliferation and function of the intestinal epithelium. This in vitro model more accurately reflects the cellular and molecular characteristics of the human colonic epithelium compared to traditional cell lines, making it a valuable tool for studying colorectal cancer biology [47]. In particular, the organoid model allows for a detailed evaluation of the response of epithelial cells to therapeutic agents, paving the way for future research focusing on identifying compounds that can inhibit ox-LDL-induced proliferation and reduce ROS production in intestinal epithelial cells.

The ability of organoids to recapitulate the heterogeneity of the colonic epithelial tissue also makes them especially useful for testing the efficacy and mechanism of action of antineoplastic drugs, as well as evaluating their impact on the dynamics of the tumor microenvironment [12,48]. Therefore, the use of colonic organoids could revolutionize the development of personalized therapeutic strategies for colorectal cancer, providing a preclinical model that allows the identification of new therapeutic targets and the screening of treatments that reduce ox-LDL-mediated cellular proliferation and oxidative stress.

This study has some limitations. First, we did not directly measure the expression levels of ox-LDL receptors (LOX-1, MSR1, CD36) after ox-LDL induction, which could have provided more conclusive evidence regarding their role in the observed effects. Second, we utilized an in vitro organoid model, which, while more representative than traditional cell lines, does not fully capture the complexity of the in vivo environment, such as interactions with the immune system.

## 4. Materials and Methods

### 4.1. Organoids

Human colonoids were grown embedded in Cultrex Reduced Growth Factor Basement Membrane Extract (R&D System, 3433-005-01) with WRN complete medium, which consisted of 50% conditioned medium (CM) containing wnt3a, R-spondin-3, and noggin [49,50]. For ox-LDL treatment, RENC medium was used [51]. The complete composition of culture media is listed in Table S1. Colonoids used were described previously [51].

### 4.2. EdU Incorporation and Click-Chemistry

Organoids were seeded on coverslips in WRN complete medium for 72 h before replacement with RENC medium and challenged with 80 μg/mL of ox-LDL for 96 h. Treatment was replaced after the first 48 h adding the same amount of ox-LDL in RENC medium. On the last day, the organoids were pulsed with 2 μM of 5-ethyl-2′-deoxyuridine (EdU) for 2 h and fixed with 4% paraformaldehyde plus 1% glutaraldehyde for 30 min. Fixed organoids were washed 3 times with TBS, 0.5% of Triton X-100, and 0.1% of Tween 20 for 5 min and 3 times with glycine 100 mM. Incorporated EdU was detected by click-chemistry reaction, incubating for 30 min with 100 mM Tris-HCl, 1 mM CuSO4, 10 μM of azide fluor 545 (Sigma 760757, Saint Louis, MO, USA), and 100 mM of ascorbic acid [52]. Coverslips were washed, and nuclei were stained with 1 µg/mL of 4′,6-diamidino-2-phenylindole (DAPI) for 10 min.

### 4.3. Measurement of Hydrogen Peroxide Production

To measure the real-time kinetics of H_2_O_2_ production from colonoids, we used a modified Amplex Red assay [53]. Organoids seeded on a 96-well plate were treated with ox-LDL for 24 h and then assayed in Dulbecco’s phosphate-buffered saline solution containing Ca^2+^, Mg^2+^, 0.1 U/mL horseradish peroxidase (Sigma-Aldrich, P8125-5KU, Saint Louis, MO, USA), 100 µM PMSF (Sigma-Aldrich, P7626), and 30 mM Amplex Red (Biotium, 10061, Fremont, CA, USA). Fluorescence was read at 120 s intervals for 1 h at 37 °C (Ex 530 nm/Em 590 nm) in a Synergy HTX (BioTek, Santa Clara, CA, USA). H_2_O_2_ production was normalized to cell viability via MTT assay. After H_2_O_2_ production was measured, the reagents were removed, and the organoids were incubated with 100 µL of DMEM/F12 (ThermoFisher, 21041025, New York, NY, USA), supplemented with 0.5 mg/mL of 3-(4,5-dimethylthiazol-2-yl)-2,5-diphenyltetrazolium bromide (MTT) (Cayman Chemical, 21795, Ann Arbor, MI, USA) for 1–2 h at 37 °C to assess cell viability. This assay quantifies metabolically active cells that reduce MTT to formazan crystals. After incubation, the formazan crystals were solubilized using 10% SDS (*w*/*v*), and absorbance was measured at 570 nm. To quantify the H_2_O_2_ production, a standard curve of H_2_O_2_ was prepared freshly for each experiment. Relative fluorescence units obtained in the Amplex Red assay were interpolated against this standard curve to calculate the amount of H_2_O_2_ produced. Absorbance values were used to normalize H_2_O_2_ production to viable cell numbers. All measurements were performed in triplicate.

### 4.4. Imaging

Organoid area: To fully capture the Matrigel dome, overlapping images were taken with the 4× objective on a Nikon Eclipse TS100 inverted microscope and a Nikon DS-Ri1 camera (Nikon Corporation, Tokyo, Japan). These images were stitched using ImageJ, v1.53c. Organoid areas were computed using the same software by manually outlining each organoid.

Edu staining: Pulsed organoids were cleared for 3 days with increasing sucrose solutions (20, 40, 80, 100, and 120%, *p*/*v*) and mounted on slides. Images were taken with a confocal microscope LSM 780 (Zeiss, Oberkochen, Germany) at multiple depths of focus using the ZEN 2011 software (Zeiss, Oberkochen, Germany). For each organoid, the total cell number and EdU+ cell number were counted using Image J.

### 4.5. RNA Purification and qRT-PCR

Organoids seeded in 24-well plates were treated with proinflammatory stimuli for 24 h. We either exposed the organoids to a mixture of 100 ng/mL of interferon-gamma (IFNγ) and 10 ng/mL tumor necrosis factor-alpha (TNFα) or to 10^7^ colony-forming units of heat-killed adherent-invasive *E. coli* (AIEC). RNA from organoids was purified using the phenol-chloroform extraction method [54]. Reverse transcription reactions were performed with the PrimeScript RT reagent Kit (Takara Bio, Inc., Shiga, Japan) using 500 ng of RNA. The cDNA was amplified on a LightCycler 480 II instrument (Roche Diagnostics Ltd., Forrenstrasse, Switzerland) using SYBR Premix Ex Taq (Takara Bio, Inc., Shiga, Japan). The sequences of the primers used are shown in Table S2.

Baseline mRNA levels for *OLR1* (LOX-1), *MSR1*, and *CD36* were determined by the delta cycle threshold (ΔCT) method, where CT represents the cycle at which the detected signal is significantly above the background signal and ΔCT is the difference between the CT of the genes of interest and the CT of the endogenous control gene, ACTB. Genes were categorized as highly expressed (ΔCT ≤ 5 cycles), intermediate (5 < ΔCT ≥ 15 cycles), low (ΔCT > 15 cycles), and undetectable (ΔCT ≥ 40 cycles) [55]. Relative expression levels of mRNA were calculated using the ΔΔCT method and normalized to the geometric mean of the housekeeping genes *ACTB* and *GUSB*.

### 4.6. Statistical Analysis

All data analysis and plots were performed using GraphPad Prism version 10 and compared using the Student *t* test, Mann–Whitney test, 2-way ANOVA, and Kruskal–Wallis test, as indicated in the legend of figures. Results are presented as mean values and SD, and a *p*-value of <0.05 was considered significant. The results are represented as mean ± SD.

### 4.7. Ethics Approval and Consent to Participate

The acquisition and use of human data and biopsies were approved by the University of Miami, Miller School of Medicine Institutional Review Board (protocol code: 20081100, date of approval 1 July 2009), and the Scientific Ethic Committee of Servicio de Salud Concepción, Chile (protocol code: 20-05-17, date of approval 7 October 2020).

## 5. Conclusions

Taken together, our findings highlight the role of ox-LDL in intestinal epithelial cell proliferation. Further elucidation of the specific receptors, intracellular pathways, and effector proteins involved in ox-LDL-mediated ROS signaling are crucial for understanding its role in neoplastic progression associated with chronic inflammation.

## Data Availability

The original contributions presented in the study are included in the article/Supplementary Materials, further inquiries can be directed to the corresponding author.

## Supplementary Materials

The following supporting information can be downloaded at: https://www.mdpi.com/article/10.3390/ph17111466/s1, Table S1: Composition of culture media for human colonoids, Table S2: Polymerase chain reaction primers. Ref. [56] is included in Supplementary Materials.
