# Supplementary material for: Oxidized Low-Density Lipoprotein Induces Reactive Oxygen Species-Dependent Proliferation of Intestinal Epithelial Cells
Source: Pharmaceuticals (Basel). 2024 Nov 1;17(11):1466. doi: 10.3390/ph17111466 (PMC11597178; doi:10.3390/ph17111466)
Supplement: Supplementary file 1 [file pharmaceuticals-17-01466-s001.zip › pharmaceuticals-3253196-supplementary.pdf]

Table S1. Composition of culture media for human colonoids

REAGENT	WRN complete	RENC
Advanced DMEM/F12	50 % vol	80 % vol
WRN-CM	50 % vol	−
Noggin-CM	−	10 % vol
R-spondin-CM	−	10 % vol
Glutamax	1x	1x
B27	1x	1x
Nicotinamide	10 mm	10 mm
EGF	50 ng/ml	50 ng/ml
Gastrin **I**	10 nm	10 nm
A 83-01	500 nm	500 nm
SB202190	10 µm	10 µm
Chir99021	5 µm	5 µm
Primocin	100 µg/ml	100 µg/ml
Thiazovivin[&]	10 µm	−

& Used in the first 48 hours after cell plating

Table S2. Polymerase chain reaction primers

Gene	Sense	Primer sequence
ACTB	Forward	TGTACCCTGGCATTGCCGACAG
	Reverse	ACGGAGTACTTGCGCTCAGGAG
GUSB	Forward	CCGATTATCCAGAGCGAGTATG
	Reverse	CTCAGCGGTGACTGGTTCG
CD36 [1]	Forward	AAGCCAGGTATTGCAGTTCTTT
	Reverse	GCATTTGCTGATGTCTAGCACA
MSR1 [1]	Forward	GCAGTGGGATCACTTTCACAA
	Reverse	AGCTGTCATTGAGCGAGCATC
OLR1 [1]	Forward	TTGCCTGGGATTAGTAGTGACC
	Reverse	GCTTGCTCTTGTGTTAGGAGGT

References

[1] Wang D, et al. C/EBPdelta-Slug-Lox1 axis promotes metastasis of lung adenocarcinoma via oxLDL uptake. Oncogene 2020;39(4):833-48.
